# Musculoskeletal Manifestations of Sickle Cell Disease: A Review

**DOI:** 10.7759/cureus.358

**Published:** 2015-10-20

**Authors:** Raju Vaishya, Amit Kumar Agarwal, Edwin O Edomwonyi, Vipul Vijay

**Affiliations:** 1 Orthopaedics, Indraprastha Apollo Hospital; 2 Orthopaedics, Irrua Specialist Teaching Hospital

**Keywords:** sickle cell disease, orthopaedic complications, surgery, avascular necrosis

## Abstract

Sickle cell disease (SCD) is an inherited disorder of abnormal haemoglobin commonly encountered in the West African sub-region. It has varied osteoarticular and non-osseous complications that mimic some surgical conditions. The most common orthopaedic complications include avascular necrosis, osteomyelitis, septic arthritis, etc. A cautious and painstaking evaluation is required in handling these patients. Acute care and anaesthetic precautions are vital in ensuring an uneventful postoperative period.

## Introduction and background

Sickle cell disease (SCD) is a hereditary disorder of abnormality in the oxygen-carrying haemoglobin molecule in red blood cells (RBC). It is inherited as an autosomal recessive disorder [1]. Morphological expression depends on the acquisition of two abnormal allelomorphic genes related to haemoglobin formation. SCD occurs when a person inherits two abnormal copies of the haemoglobin gene, whereas a person with a single abnormal copy is said to have the 'sickle-cell trait' and they usually do not experience symptoms. It has no sexual predominance and is a lifelong disease. A study of the natural history of SCD indicates that about 5% of sickle cell patients’ account for nearly one-third of the hospital admissions. Its diverse manifestations need to be differentiated from other surgical conditions [2]. Some of its consequences require orthopaedic care and, therefore, stimulates orthopaedic interest. These patients need to be thoroughly assessed as there are anaesthetic as well as perioperative risks involved. The medical condition of the patient should be optimised. This article highlights the typical orthopaedic complications of the disease as well as the challenges associated with the management.

### History

**Figure 1:**
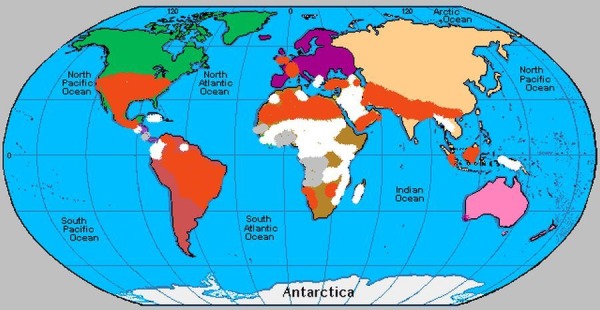
Global prevalence of sickle cell disease.[Orange: 0.1-0.99, White: 1- 9.9, Grey: 10- > 19 Births with pathological Hb disorder per 1000 live birth]

The sickle-cell trait originated in West and Central Africa centuries ago. From there, the gene spread along the Mediterranean, the Persian Gulf, parts of India, and across the Atlantic. Recently, it has spread more widely in Europe but is rarely encountered south of the equator. The slave trade carried SCD to North America, the Caribbean, Central America, and a few countries of South America. More recent migration from Africa and the Caribbean has brought it to the British Isles. The incidence in United States blacks is 8%, 10% in northern Ghana, 30% in Northern Nigeria, and 2 - 45% in East Africa [3]. Figure 1 below shows the global prevalence of SCD.

In 1910, a Chicago cardiologist, Professor James Herrick, described the sickle cell in the blood film of a dental student, Walter Clement Noel [4]. In 1922, a medical student, Verne Mason, at John Hopkins University coined the term sickle cell anaemia [5]. Some 50 years earlier, the African medical literature was quoted to have reported the condition. Long before this time, this condition was known amongst the lgbos in Southeast Nigeria and Binis in the South Nigeria as Ogbanje and Igbakhwan, respectively. There was recognition that the children involved died early, and it was assumed that they came to bring ill luck and pain to their families. Curiously, more than one child in the family usually had it, with series of ill health and subsequent death. In 1949, Linus Pauling, et al. were the first to demonstrate that SCD occurs as a result of an abnormality in the haemoglobin molecule. They were the first to link it as a genetic disease due to a mutation of a specific protein [6]. In 1956, Ingram revealed that it was the substitution of valine for the glutamic acid in the sixth position of the beta globulin molecule that was responsible for the abnormal function of the molecule after deoxygenation [7].

## Review

### Pathophysiology

The normal adult haemoglobin is made up of two alpha and two beta globulin chains in combination with a heme molecule, making up 95% of the normal human globulin. Variants of the structural haemoglobin are based on a point mutation in a globin gene that produces a single amino acid substitution in a globin chain [8]. The mutation substituting thymine for adenine in the 6^th^ codon of the beta chain leads to the development of the haemoglobin S (HbS). This results in the substitution of amino acid valine for the normal glutamic acid at the position 6 of the beta chain.

Under deoxygenation, HbS polymerizes, leading to initial reversible structural changes. It ultimately leads to permanent membrane damage after repeated deformation. Infection, physical exercises, acidosis, cold, and dehydration are other conditions that predispose HbS to polymerization. Following deoxygenation, HbS become fragile with marked decreased solubility and elasticity. These abnormal sickle cells fail to return to normal shape when there is a restoration of oxygen tension.

**Figure 2:**
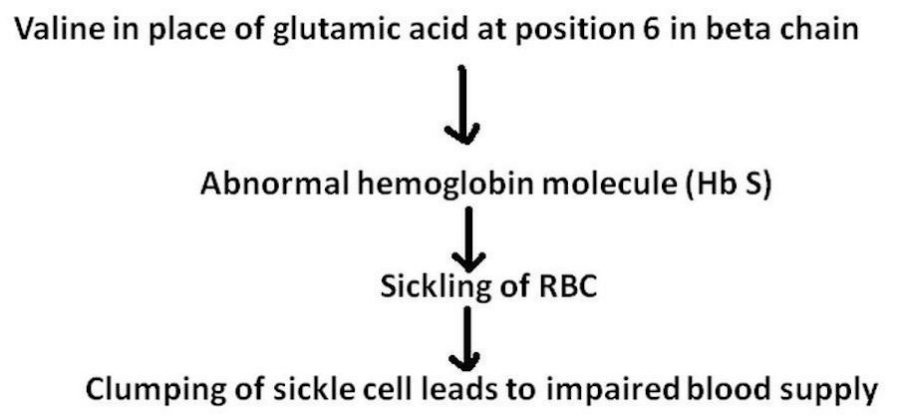
Pathophysiology of sickle cell disease

Red cells containing high levels of sickle haemoglobin contribute to the pathophysiological development of sickle cell anaemia in three ways [9]. Firstly, the loss of deformability leads to vascular obstruction and ischemia. This critical factor is responsible for acute chest syndrome (ACS), bone crises, functional asplenia, and acute stroke. Secondly, membrane damage shortens the life span of the red cell resulting in haemolysis, both intra- and extravascular. The intravascular haemolysis contributes to decreasing the availability of nitrous oxide, increased vascular tone, and pulmonary artery hypertension. Lastly, damaged red cells have an abnormal surface that leads to increased adherence to and damage of the vascular endothelium. This process enhances vascular obstruction and also provokes a proliferative lesion involving white cells, platelets, smooth muscle cells, cytokines, growth factors, and coagulation proteins. Figure 2 briefly explains the pathophysiology of SCD.

Abnormalities in coagulation, white cells, vascular endothelium, and damage to the membranes of red cells are other aspects critical to the pathophysiology of this disease. Increased leukocyte adherence to the endothelium also plays a role in the pathogenesis of vascular occlusion. As a result, patients develop both haemolytic anaemia and vasculopathy [10]. Organ damage is an ongoing problem with multi-organ infarcts. Although episodes of pain punctuate the disease course, the organ damage is often silent until it is far advanced [11]. While healthy red cells live up to 90-120 days, sickle cells only survive 10-20 days due to sequestration by the spleen as a result of their abnormal shapes, giving rise to haemolysis. The summary of the above pathogenesis is a clinical condition that can lead to a haemolytic sequestration and aplastic crisis, along with multi-organ infarcts that culminate in a series of orthopaedic and surgical complications. Table 1 below lists the common orthopaedic problems encountered in SCD.

**Table 1:** Common orthopaedic complications encountered in sickle cell disease

	Orthopaedic complications
1	Avascular necrosis of hip
2	Osteomyelitis
3	Septic arthritis
4	Leg ulcer
5	Growth retardation and skeletal immaturity
6	Dactylitis
7	Osteoporosis and vertebral collapse
8	Pathological fracture
9	Arthritis

### Specific orthopaedic complications

Avascular Necrosis and Medullary Infarct

**Figure 3:**
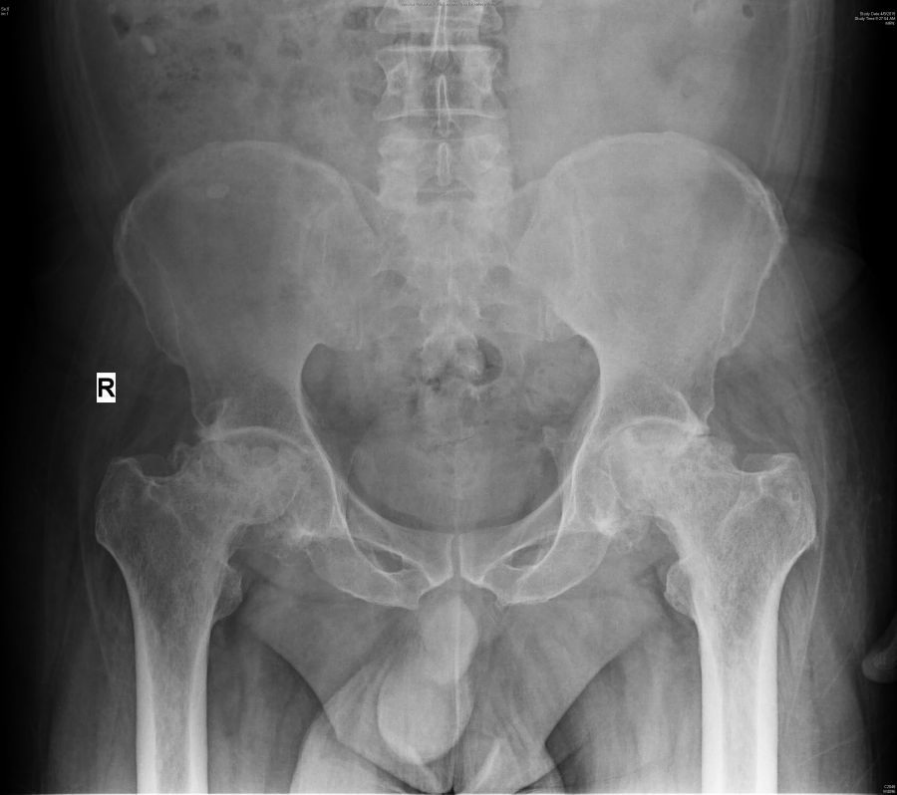
X-ray pelvis anteroposterior view showing avascular necrosis of the head of the femur

Avascular necrosis (AVN) of the bone is a relatively common problem among patients with SCD. The femoral head is the most common area of bone destruction in those patients (Figure 3) [12].

**Figure 4:**
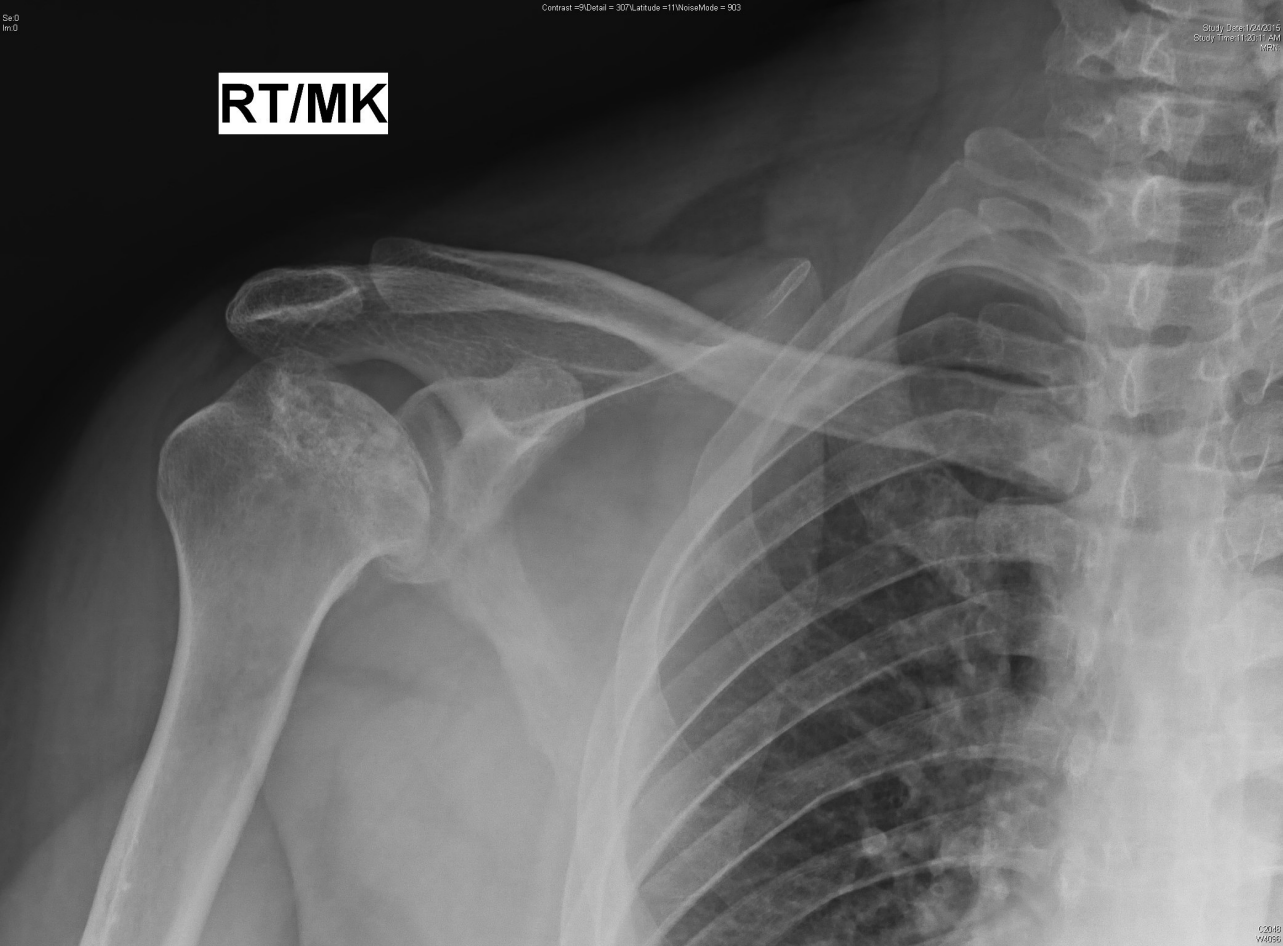
X-ray right shoulder anteroposterior view showing avascular necrosis of the head of the humerus

**Figure 5:**
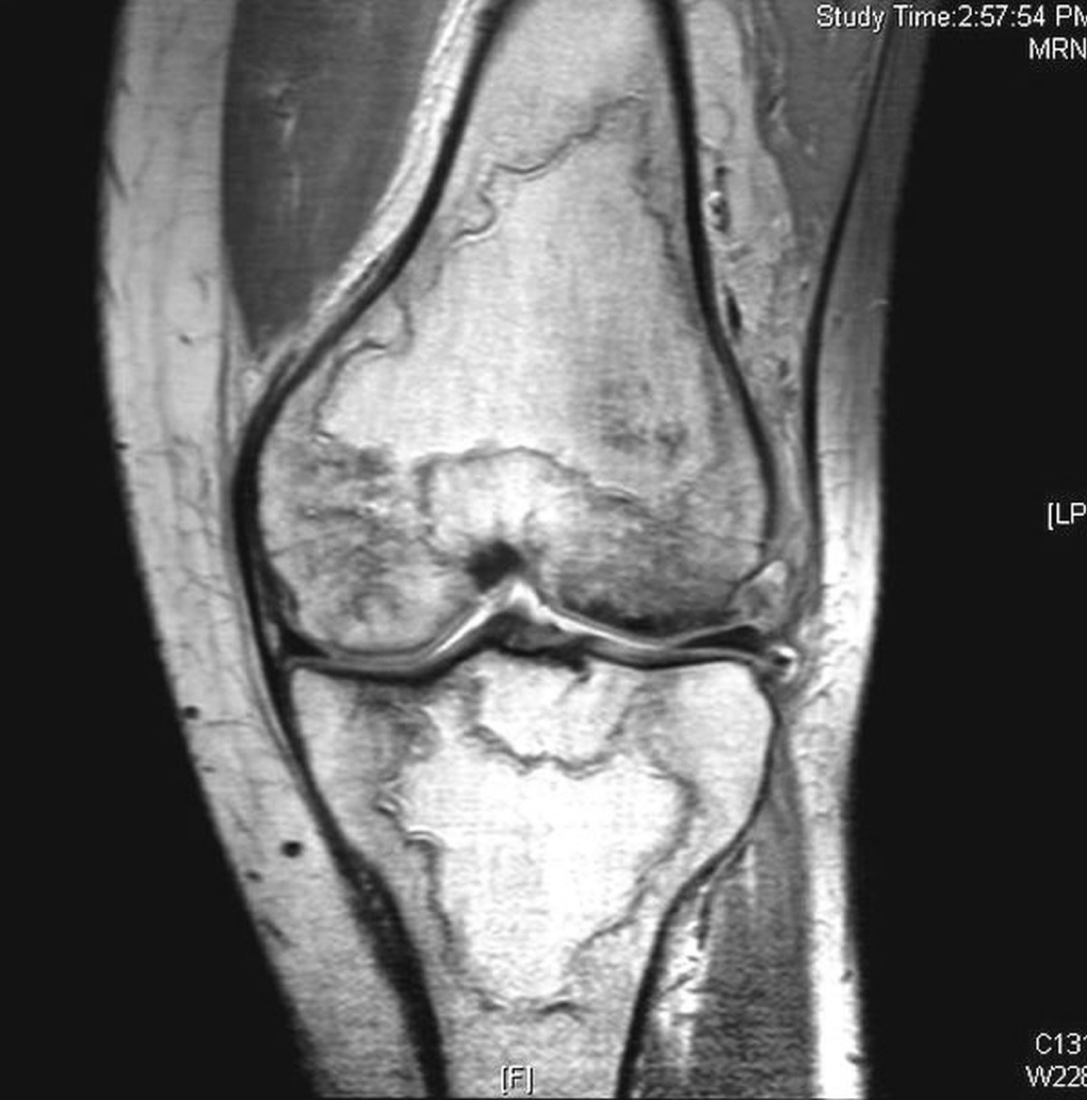
MRI of the knee showing multiple infarcts

Other sites affected are the shoulder, knee, and talus as shown in Figures 4-5.

AVN in SCD is different from AVN due to other causes. In SCD, the entire epiphysis of the long bone is uniformly affected while, in other conditions, the weight-bearing areas are commonly involved. The initial infarcts often occur in the subchondral regions where the collateral circulation is minimal. The overlying articular cartilage often remains viable in the early stages of the disease because it receives nutrition from the synovial fluid. If the pace of the “creeping substitution” within the subchondral infarct is too slow to be effective, then micro-fractures develop that eventually lead to the collapse of necrotic cancellous bone and ultimately to joint destruction [13]. This almost always includes the anterior superior portion of the proximal femur, where the weight-bearing forces are the greatest [14]. Early reports have found a higher rate of AVN in homozygous SCD compared with other sickle mutations. However, recent studies have not been able to find any significant difference between the Hb SS or Hb SC genotype [15-16]. These studies have reported several mutations, unrelated to the haemoglobin disorder, which influence the severity of the disease by altering the genes involved in its pathophysiology [17].

The pathogenesis of AVN is still not clear. Several possible factors have been implicated, namely, intravascular coagulation, microvascular occlusion (following marrow hyperplasia with tissue crowding), fat embolism, intraosseous stress, and immunologic factors. The commonest presentation of AVN of the hip includes pain, limitation of movements, and limp. Plain x-ray features of AVN are attenuation of the epiphysis, lucent areas, flattening or collapse of articular surfaces, sclerosis, and joint space narrowing. However, these signs are only seen in advanced disease. Magnetic resonance imaging (MRI) is a useful tool for detecting and grading early changes [18].

Various classification methods to grade AVN of the femoral head exist. The Arlet, Ficat, and ARCO (Association Research Circulation Osseous) are some of the commonly used classifications [19-20]. The treatment of early disease may require prolonged bed rest with the traction of the affected limb to relieve the weight, physical therapy, and subsequently graduated weight-bearing. Untreated asymptomatic AVN of the femoral head in patients with SCD has a high likelihood of progression and collapse. Therefore, early surgical intervention to retard the progression of the disease is helpful [21]. Many surgical procedures have been proposed to treat AVN, but none is entirely satisfactory. Core decompression with the removal of necrotic zones and concomitant bone grafting or muscle pedicle grafting is a vital option for early cases. The purpose of core decompression is to promote osteogenesis and improve bone repair. Electrical stimulation may be transiently effective in some cases. Autologous bone marrow graft or acrylic cement may be helpful to support the subchondral bone at risk of collapse [22-23].  The procedure of the core decompression with a vascularized fibular transplant is a promising treatment for AVN, but it has not been evaluated in those who have SCD. Unfortunately, it does not completely alleviate pain and is associated with a longer rehabilitation period, both of which are disturbing to those who have sickle cell disease. Arthroscopic and surgical debridement are other options available. Upper femoral osteotomy redirects the load on the femoral head and prevents further collapse of the femoral head. Innominate osteotomy may be indicated in cases of joint subluxation. Total hip arthroplasty (THA) is the only option left with advanced stage disease [24]. Overall, patients with SCD requiring THA are younger than the general population.

Osteomyelitis

Nearly 90% of SS sicklers suffer from osteomyelitis of one or more bones before they are ten years of age. No bone is exempted from osteomyelitis, and several bones could be affected at a time. Hyposplenism, impaired complement activity, and the presence of infarcted and necrotic bone are a few factors that predispose them to infections. The common infective agents include Staphylococcus pyogenes and coliforms. Salmonella typhi is more common in SCD patients than in the general population [25-26]. No long bone is exempted. It could be multifocal in some cases. Osteomyelitis commonly affects the diaphysis of the femur, tibia, or humerus, whereas the metaphyseal location is more prevalent in the non-sickle population [27].

The acute attack of bone infarction could present as persistent fever, swelling, bone tenderness, leucocytosis, and septicaemia. Like acute osteomyelitis, significant x-ray finding is usually not seen in the early stages of bone infarction. Therefore, differentiating acute osteomyelitis from infarction may be difficult in an acute attack [28]. Infarction can be 50 times more common than bacterial osteomyelitis in those who have sickle cell disease [29]. It is admissible, however, that a persistent leukocytosis with fever, overabundant involucrum suggests osteomyelitis. Radionuclide scanning can be useful. It has been shown that a combination of the 99mTc-sulphur colloid and 99mTc-diphosphonate or 99mTc with gallium leads to improved accuracy [30]. The marrow uptake is normal in osteomyelitis but increased in infarction. The pitfall of this investigation is that both false positives and false negatives do occur. Radiolabelled leukocyte scans similarly fail to reliably discriminate between osteomyelitis and infarction [31]. MRI shows considerable overlap between infection and infarction. However, specificity is increased by contrast enhancement. It is more useful in the localization of the lesion and monitoring of response to treatment. Therefore, despite the progress made in the development and use of imaging techniques, a definitive diagnosis of acute osteomyelitis in SCD still relies more on clinical assessment together with positive cultures from blood or bone obtained by aspiration or biopsy than upon any single imaging modality.

Treatment of acute osteomyelitis is mostly conservative and may require immobilization in plaster or traction, analgesics, and broad-spectrum antibiotics for six weeks. Chronic osteomyelitis may manifest as a discharging sinus. The infection is mostly contained locally, and systemic signs are common. An x-ray may reveal sequestrum, abundant involucrum, and cloaca. Wound swab culture and biopsy are advised. Treatment involves adequate surgical debridement, management of the resultant dead space, and antibiotic coverage based on a sensitivity pattern. Depending on the site of the osteomyelitis, orthotics or non-weight bearing may be indicated.

Septic Arthritis

Septic arthritis (SA) results from vaso-occlusion involving articular surfaces with subsequent infection [32]. It is usually by haematogenous spread. The profile of the organisms is similar to that of osteomyelitis. Diagnosis can be confirmed by a culture of joint aspirate. Early diagnosis and treatment are essential to prevent irreparable joint damage. The treatment entails bed rest, drainage, and splinting of the joint, graduated mobilization, analgesics, and antibiotics. Arthroscopic debridement and irrigation are rewarding.

Leg Ulcers

SCD patients commonly present with leg ulcers with up to 90% occurring in the lower third of the leg above the medial malleolus.

It is bilateral in 30% of the cases, and it is usually superficial. The cause is multifactorial and has been associated with trauma, infection, anaemia, and circulatory defects in the ankle due to stasis and thrombosis. Treatment includes bed rest, limb elevation, dressings, compression bandaging, and use of zinc tablets and zinc cream. Ulcer excision and subsequent skin cover with skin graft provide a good result. A split skin graft is prone to excessive fibrosis and recurrence of an ulcer. A human epidermal autograft is being advanced.

Growth Retardation and Skeletal Immaturity

**Figure 6:**
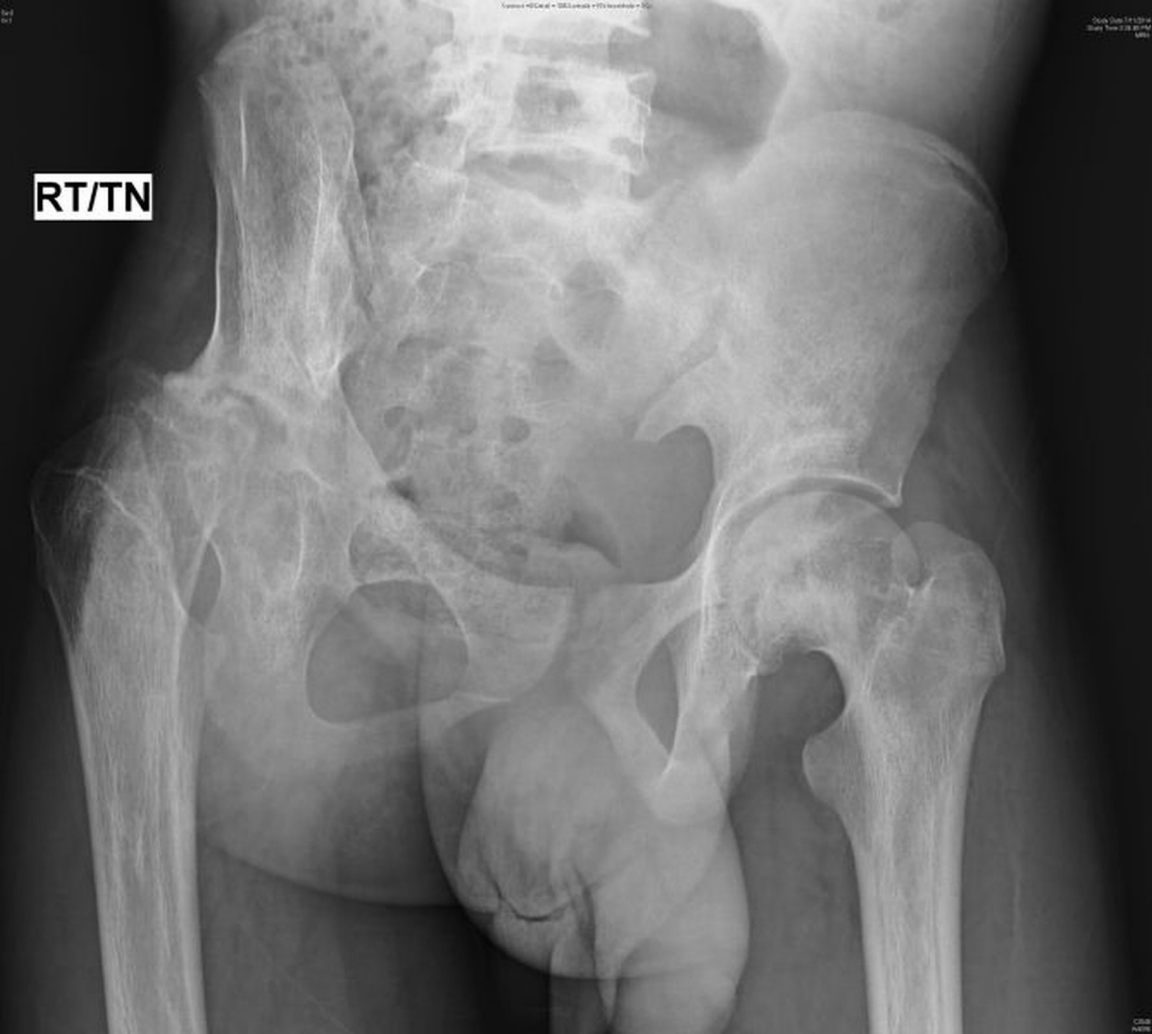
X-ray pelvis anteroposterior (AP) view showing retardation in growth of the proximal femur

Abnormalities of growth are one of the primary orthopaedic complications of SCD in children. The pathological basis for these is numerous. Marrow hyperplasia leading to ischaemia accounts for the H-shaped and ‘tower’ vertebrae of the spine. Local anoxic events may result in the premature closure of the epiphysis and impaired or even asymmetrical growth of the long bones of the limbs. Skeletal immaturity and subsequent retardation in growth and angular deformities are usually seen (Figure 6).

The ongoing hematopoietic activity involving the skull bones leads to expansion and subsequent frontal bossing observed in some of these patients.

Dactylitis

Hand and foot syndrome is common at six months to seven years of age. These are symptomatic microinfarcts in the phalanges and metatarsal bones, although there is a report of dactylitis developing in a 26-year-old who had HbS/β-thalassemia [33]. Symptoms of dactylitis consist of unilateral or bilateral painful swelling of the distal extremities. This painful swelling of the hands and feet is often associated with leukocytosis and fever. The symptoms mimic osteomyelitis or cellulitis, but unlike these conditions, the symptoms of dactylitis are self-limiting and usually resolve within a month [34].

Histologically, there is an extensive infarction of the marrow, medullary trabeculae, and an inner layer of the cortical bone, together with the subperiosteal new bone formation. In two to three weeks, an x-ray may show a ‘moth-eaten’ appearance of the involved digits. This is due to cortical thinning and irregular attenuation of the medullary spaces. Conservative treatment is advised since symptoms usually resolve within weeks. Treatment includes bed rest, elevation, immobilization, and analgesics. Mild heat is acceptable, but avoidance of ice or cold is necessary to avoid a vaso-occlusive crisis [35].

Osteoporosis and Vertebral Collapse

Patients with SCD have been shown to have a lower bone density at all sites compared with age match in the general population [36]. This is attributed to erythroid hyperplasia due to increased haemopoietic activity in the bone resorption. This leads to generalised osteoporosis and thinning of the cortex, especially in the vertebrae. Radiologically, the vertebral body appears as biconcave or ‘fish-mouth’ due to the effect of the intervening disc on the adjoining vertebral end plate. Vertebral collapse can thus readily occur following weakening from thrombosis or infarction. This is common sequelae to osteoporosis involving the vertebrae. The vertebral changes consist of central deterioration with preserved peripheral areas of the vertebral end-plates. This core destruction is a result of the anatomic blood supply to the end-plates. The central portion of the vertebral end-plate derives its blood supply from the long branches of the vertebral nutrient artery while the peripheral part of the end-plate is provided by short perforating branches of the periosteal vessels. The longer end arterial vessels are more likely to exhibit vaso-occlusion and destructive events. Rest or physical therapy is often only minimally helpful, but a proper thoracic or lumbar orthosis can be beneficial and diminish the need for medications. Surgical intervention may be required in advanced collapse.

Pathologic Fracture

Osteoporotic bone is prone to pathologic fracture as it cannot withstand normal physiological stresses. Repeated infarcts give rise to areas of necrosis and weakening that are prone to pathologic fractures. These weakened bones can be protected by external casts and fractures fixed internally or externally as the case may require.

Arthritis

Several forms of arthritis, both inflammatory and non-inflammatory, are described in association with SCD, including bone infarcts, hyperuricemia, gout, and osteomyelitis. It progresses to destruction of the critical joint components and bone erosion [37]. Cases of synovitis, with infiltration of plasma cells and evidence of joint destruction, have also been described in SCD [38]. Arthritis associated with SCD is usually polyarticular (more than 80% of cases) and symmetric (over 60% of cases), with a predilection for large joints and lower extremities and generally lasting less than a week [39]. X-rays can identify periarticular osteopenia, bone erosion, synovitis, and joint space narrowing.

## Conclusions

Sickle cell disease is a genetic disorder with multisystem involvement. Musculoskeletal affection accounts for much of the morbidity suffered by these patients. The main radiological changes seen in bone are secondary to hyperplasia of the marrow and ischemic osteonecrosis. Vaso-occlusive crises of sicklings are difficult to differentiate from acute infections of the musculoskeletal system. Recent advancement in the diagnostic modalities using isotope scans and magnetic resonance imaging are helpful in obtaining an accurate diagnosis. If there is osteomyelitis, a high index of suspicion for concomitant acute septic arthritis is required. Aggressive medical management of painful crisis and maintenance of a stress-free environment is desirable to minimize morbidity in these patients. Reconstructive and replacement surgical procedures for joint destruction offers a fair prognosis for patients affected with the sickle-cell disease. Efforts should be channelled towards prevention as well as dealing with these complications by thorough and painstaking evaluation and monitoring during regular clinic visits as well as during the perioperative period. Surgeons should not fail to make it a point of duty to inform patients of the risks involved in the surgical intervention, and surgeons must be prepared to deal with the many possible complications.
